# Interaction preferences between protein side chains and key epigenetic modifications 5-methylcytosine, 5-hydroxymethycytosine and N^6^-methyladenine

**DOI:** 10.1038/s41598-022-23585-z

**Published:** 2022-11-15

**Authors:** Matea Hajnic, Santiago Alonso-Gil, Anton A. Polyansky, Anita de Ruiter, Bojan Zagrovic

**Affiliations:** 1 Department of Structural and Computational Biology, Max Perutz Labs, University of Vienna, Campus Vienna Biocenter 5, 1030 Vienna, Austria; 2 Present Address: Bayer AG, Computational Life Sciences, Research & Development, Crop Science, Frankfurt, Germany; 3 Present Address: Institute of Molecular Modeling and Simulation, BOKU, Muthgasse 18, 1190 Vienna, Austria

**Keywords:** Computational biophysics, Chemical modification

## Abstract

Covalent modifications of standard DNA/RNA nucleobases affect epigenetic regulation of gene expression by modulating interactions between nucleic acids and protein readers. We derive here the absolute binding free energies and analyze the binding modalities between key modified nucleobases 5-methylcytosine (5mC), 5-hydroxymethylcytosine (5hmC) and N^6^-methyladenine (m^6^A) and all non-prolyl/non-glycyl protein side chains using molecular dynamics simulations and umbrella sampling in both water and methanol, the latter mimicking the low dielectric environment at the dehydrated nucleic-acid/protein interfaces. We verify the derived affinities by comparing against a comprehensive set of high-resolution structures of nucleic-protein complexes involving 5mC. Our analysis identifies protein side chains that are highly tuned for detecting cytosine methylation as a function of the environment and can thus serve as microscopic readers of epigenetic marks. Conversely, we show that the relative ordering of sidechain affinities for 5hmC and m^6^A does not differ significantly from those for their precursor bases, cytosine and adenine, respectively, especially in the low dielectric environment. For those two modified bases, the effect is more nuanced and manifests itself primarily at the level of absolute changes in the binding free energy. Our results contribute towards establishing a quantitative foundation for understanding, predicting and modulating the interactions between modified nucleic acids and proteins at the atomistic level.

## Introduction

Modifications of standard DNA/RNA nucleobases greatly amplify the amount of information encoded in nucleic-acid sequences and are associated with epigenetic regulation of gene expression and modulation of transcript stability^1–3^. In DNA, modified nucleobases play key roles in cell differentiation, aging and disease development by either remodeling chromatin structure or affecting directly the DNA/protein interactions^1,2,4–6^. In RNA, nucleobase modifications affect different molecular processes, including mRNA transcription, splicing, export, translation and degradation^3,7–10^. Notably, the impact of the DNA/RNA modifications on gene expression and transcript stability has mostly been studied from the perspective of how the change in their genomic or transcriptomic patterns affects the cellular or organismic phenotype^3,5^. However, less is known about the atomistic mechanisms behind such phenotypic changes and, in particular, how nucleobase modifications affect the interactions between the modified nucleic acids and protein readers, which detect them. In general, DNA and RNA recognition by proteins depends on different environmental, structural and dynamical determinants. Importantly, such recognition also directly depends on the intrinsic binding preferences between the individual nucleobases and amino acids in different environments^11^. While the binding preferences between standard nucleobases and amino acids have in general been well studied^11–20^, much less is known about how these preferences change in the case of typical nucleobase modifications, with most work focusing on the overall impact of modifications on the affinities between complete nucleic acids and proteins^21–24^.

**Figure 1:**
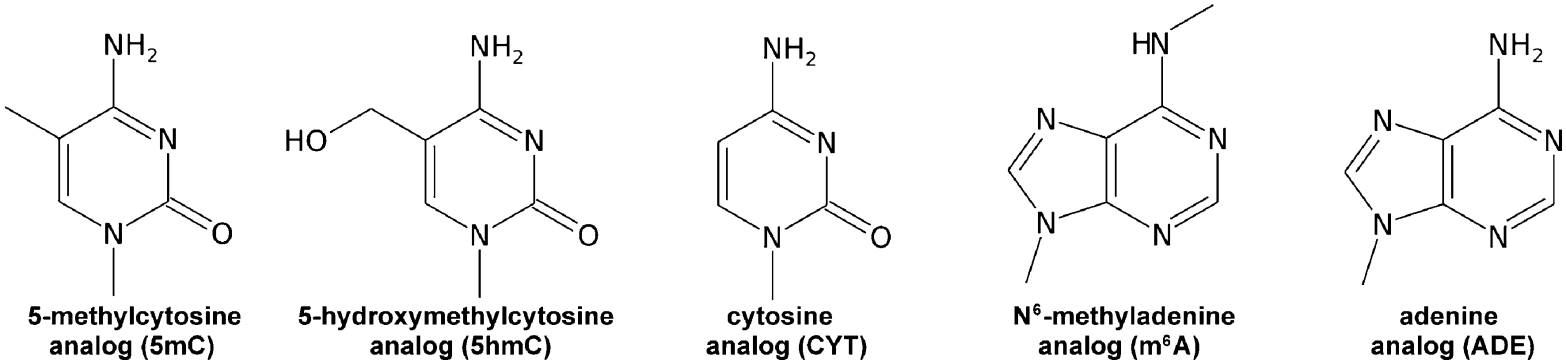
Chemical structures of 5mC, 5hmC, CYT, m^6^A and ADE analogs used in simulations.

Motivated by this, we focus here on the interaction preferences between protein side chains and three abundant and well-studied modified nucleobases with critical roles in both DNA and RNA biology: 5-methylcytosine (5mC)^5,6,25–27^, 5-hydroxymethylcytosine (5hmC)^25,28–31^ and N^6^-methyladenine (m^6^A)^3,32–35^ (Fig. 1). Specifically, 5mC, a modified derivative of cytosine (CYT), is the most abundant modification in DNA that is introduced by different DNA- and RNA-methyltransferases^6,10,36,37^ (Fig. 1). It has been estimated that ~ 4% of all DNA CYTs in mammals are modified to 5mC, while in the CpG regions this fraction increases to ~ 80%^5^. 5mC is mostly found within promoter and enhancer regions, where its occurrence is associated with gene repression^4^. In DNA, 5mC is recognized by methyl-CpG-binding domain (MBD) proteins that bind methylated CpG regions^37,38^ and recruit different histone modifiers^37,39,40^. Importantly, many transcription factors (TFs) are sensitive to CYT methylation in DNA^41^, with a caveat that trends observed in vitro do not necessarily hold in vivo^42^. Finally, in RNA, 5mC is recognized by different readers, such as ALYREF^27^ and YBX1^25^, which modulate RNA stability^10^.

In both DNA and RNA, 5mC is oxidized to another prominent modified nucleobase, 5hmC (Fig. 1), by the Ten eleven translocation (TET) enzymes^30,31,43^. 5hmC has been found in DNA promoter and enhancer regions where its occurrence correlates positively with gene expression. The abundance of 5hmC has been estimated at 0.1% of DNA cytosines in mammalian tissues^28^, with this fraction increasing up to 1% in the nervous system^5,44^. Furthermore, the number of known proteins that recognize and bind 5hmC in DNA is rapidly growing to now include TET enzymes, some transcription factors, UHRF2, MBD3 and MeCP2^5,28,30,37^. While 5mC is typically associated with the repression of transcription, its first derivative 5hmC is mostly responsible for the restoration of active transcription^5,28,30,37^. The amounts of RNA 5hmC have been found to correlate with transcript abundance^45^, but in contrast to DNA 5hmC, the nature and the functional role of readers of RNA 5hmC remain to be explored.

Finally, adenine modification m^6^A (Fig. 1) is the most frequent internal mRNA modification, with important roles in different cellular and physiological processes, ranging from splicing to translation to regulation of mRNA stability^3,10^. m6A is primarily added to mRNAs via a writer complex consisting of the core N6-adenosine methyltransferase METTL3 and its adaptors^46^, while its readers belong to a diverse group including YT521-B homology (YTH) domain-containing proteins, heterogeneous nuclear ribonucleoproteins, and insulin-like growth factor 2 mRNA-binding proteins^3,8,10,46,47^. When it comes to DNA, m^6^A has been identified in different organisms at levels of up to 0.4% genomic adenines and is introduced in DNA by writers such as methyltransferase DAMT-1 in *C. elegans* ^3,32–35,48–50^. These studies have also identified demethylation enzymes (TET homologues) whose depletion is tightly correlated with m^6^A abundance^32–34^. In mammals, m^6^A has largely been found within transposons and its occurrence has been positively correlated with their silencing and the repression of transcription from adjacent genes^33^. Finally, there are a number of eraser proteins, which recognize and remove the above DNA/RNA nucleobase modifications^3,46,51^.

While there exist hundreds of different biological relevant DNA/RNA nucleobase modifications, we focus on 5mC, 5hmC and m^6^A due to their overall abundance and biological/biomedical significance, as outlined above. Overall, our primary aim is to contribute to the fundamental understanding of the interactions between modified nucleobases and protein readers from a reductionist, physicochemical perspective. Specifically, we use atomistic molecular dynamics (MD) simulations and umbrella sampling to derive for the first time the absolute binding free energies and the associated interaction mechanisms between 5mC, 5hmC and m^6^A nucleobases and all standard, non-glycyl/non-prolyl amino-acid side chains in water and methanol. The latter is chosen as an approximation of the low-dielectric environment at nucleic-acid/protein interfaces^12,52–54^. We compare the obtained absolute binding free energies to the corresponding binding free energies of standard nucleobases (Fig. 1) derived previously^12^ and rationalize the binding mechanisms in known systems in light of the newly obtained binding affinity scales.

## Materials and methods

### Parameterization of modified nucleobases

Parameters for modified nucleobases were derived from those corresponding to the most similar nucleobases in the GROMOS 54a8 force field^55^. Specifically, the cytosine nucleobase parameters were used as a scaffold for both 5mC and 5hmC. In the case of 5mC, the parameters for the methyl group at C5 position were taken from thymine. The hydroxymethyl group parameters of 5hmC at C5 position correspond to the parameters of the serine sidechain analog. The parameters of adenine were used for m^6^A, with the only change made for the amino group at the C6 position, where one of the hydrogen atoms was replaced with the parameters taken from the peptide bond. The N^6^-methyl group was set in the plane of the base and pointing in the direction of the Watson–Crick edge i.e. m^6^A was simulated as a free (not base-paired) base. In addition, the methyl group at position N9 was added to all modified nucleobases at the site of sugar attachment. Side chains were parameterized to match the corresponding amino acids with the backbone atoms replaced by a single H-atom attached to Cβ to change it from CH_2_ to CH_3_ i.e. backbone was fully removed. As the applied force field uses a united-atom formalism, this effectively just meant that Cβ was treated as a single interacting particle with parameters of the methyl group CH_3_.

### Molecular dynamics (MD) simulations

MD simulations with umbrella sampling were used to derive the absolute binding free energies of all 18 natural amino-acid side chains (all except Gly and Pro) and 5mC, 5hmC or m^6^A in both water and methanol. In each simulation setup, a single modified nucleobase and a single amino-acid side chain were placed in a cubic simulation box with centers of geometries 2 nm apart and immersed in one of the two solvents. All simulations were carried out using the GROMACS 5.0. simulation package^56,57^ and united-atom GROMOS 54a8 force field^55^ with a 2 fs integration step. The SPC water model^58,59^ was used in water simulations with anywhere between 3074 to 3878 water molecules per box (with the length of a cubic box between 4.5 and 4.9 nm). Box sizes remained the same in methanol simulations, but the number of solvent molecules changed to 1366–1765, depending on the system. Ionizable protein sidechains were set to correspond to a pH of 7. Histidine residue was simulated in its charged (His_H_) and both neutral forms (His_A_, His_B_). All bonds were constrained using LINCS^60^, while non-bonded interactions within a 0.8 nm range were calculated based on a pairlist that was updated every 5 steps. The interactions between 0.8 and 1.4 nm were calculated only with every pairlist update and were kept constant otherwise. Interactions beyond 1.4 nm were treated via a reaction-field contribution with a dielectric permittivity of 61 for SPC water^59^ and 18.6 for methanol^61^. The temperature and the pressure were kept at 298 K using the Berendsen thermostat^58^ (τ_T_ = 0.1 ps) and 1 atm using the Berendsen barostat (τ_p_ = 0.5 ps, compressibility = 4.5 × 10^–5^ bar^−1^ in water^58,62^ or 1.25 × 10^−4^ bar^−1^ in methanol^63,64^), respectively.

Steepest descent algorithm with 25,000 steps was used for energy minimization. The equilibration was performed in six independent steps. In the first step, position restraints (with a force constant of 2.5 × 10^4^ kJ mol^−1^) were applied to solute molecules, with the initial velocities drawn from the Maxwell–Boltzmann distribution at 50 K. In the next four equilibration steps, the temperature was raised by 50 K and the force constant of the position restraints lowered by a factor of 10 at each step. In the last equilibration step, the temperature was set to 298 K, while position restraints were switched-off and center-of-mass-translation was removed every 1000 steps. The first four equilibration steps were simulated for 20 ps each, while the last step took 40 ps. All equilibration steps were performed in the NPT ensemble.

### Umbrella sampling

The absolute binding free energies of amino-acid sidechain/modified nucleobase pairs were derived from potentials of mean force (PMFs) using the methodology previously described in de Ruiter et al.^12^. Here, the PMFs were constructed using the distance between the centers of geometry of a modified nucleobase and an amino-acid side chain as the reaction coordinate. To enhance the sampling along the reaction coordinate, umbrella sampling with a force constant of 500 kJ mol^−1^ nm^−2^ was used. The restraining distances ranged from 0.4 to 1.9 nm and were changed in steps of 0.1 nm. At each step, an equilibration of 100 ps preceded a production run of 10 ns. To test for convergence, the production runs were split into two 5-ns long segments, and the PMFs and the binding free energies calculated for each. If the difference in ΔG_binding_ between the two segments exceeded 1.5 kJ/mol, the production runs for all distances were prolonged for additional 10 ns until this criterion was met.

### Analysis of amino-acid enrichment at known 5mC/CYT interfaces

A comprehensive set containing all available X-ray structures of unique nucleic-acid/protein complexes in which CYT and 5mC moieties interact directly with the same protein was obtained from the PBD. The average resolution of the 101 X-ray structures in the set (PDB codes given in the Supplementary Information) was 2.2  ± 0.4 Å, with the lowest resolution being 3.2 Å. Enrichment *E* was defined as the ratio of the total relative surface area corresponding to a given amino acid at the interface with a given nucleobase and the total relative surface area occupied by the amino acid on the whole surface. A proxy of the relative binding free energy of a given amino acid with CYT and 5mC was estimated as the negative natural logarithm of the ratio between the average enrichment of residues in direct van-der-Waals contact with 5mC or CYT i.e. −ln(*E*(5mC)/*E*(CYT)), and compared with the Factor 1 hydrophobicity scale^65^ and ΔΔG_methanol_ values. For Factor 1 comparison, amino acids were additionally grouped by their physicochemical properties as follows: apolar (Ala, Cys, Ile, Leu, Met, Val), aromatic (Phe, Trp, Tyr, His), OH-containing (Ser, Thr), amide-containing (Asn, Gln), positively charged (Arg, Lys), negatively charged (Asp, Glu), while Gly and Pro were treated individually and were not grouped with other amino acids for this analysis.

## Results

### Protein sidechain binding free energies of 5mC and 5hmC

**Figure 2:**
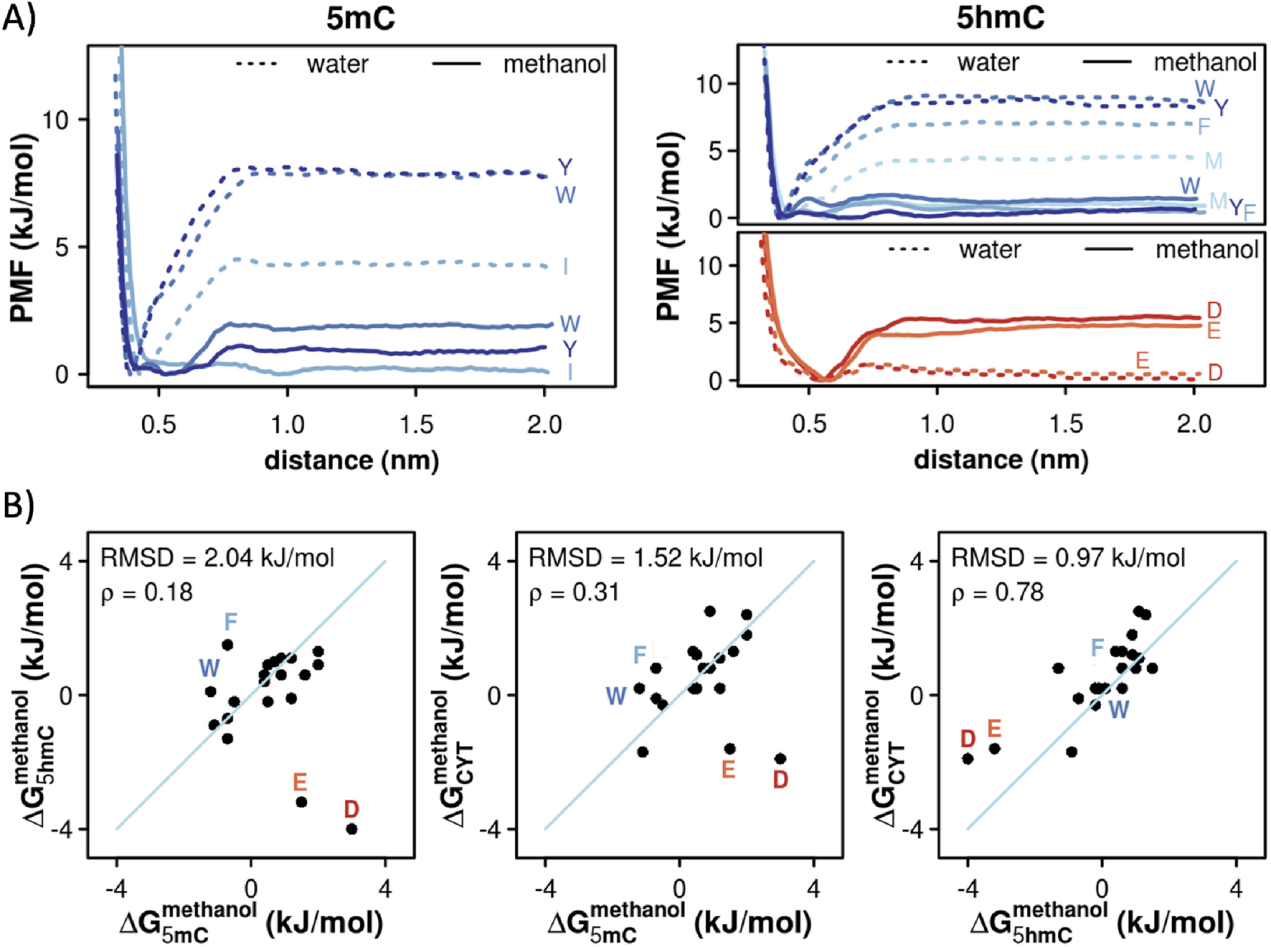
Absolute binding free energies of protein side chains for 5mC and 5hmC. (**a**) PMF curves derived for protein side chains whose affinities for 5mC (left panel) and 5hmC (right panels) change significantly (> 3 kJ/mol) depending on the surrounding solvent. (**b**) A comparison between sidechain affinity scales in methanol for: 5mC and 5hmC (left panel), 5mC and CYT^12^ (middle panel) and 5hmC and CYT (right panel) with the corresponding Spearman correlation coefficients and RMSD values. Side chains that deviate the most in the affinities for the two nucleobases being compared are labeled in color.

The potentials of mean force (PMFs) derived from MD simulations suggest that in water 5mC interacts most strongly with the aromatic side chains of Trp, Tyr and Phe, with the corresponding absolute ΔG_5mC (water)_ of −4.3 kJ/mol, −3.9 kJ/mol and −3.0 kJ/mol, respectively (Fig. 2A left, Table 1 and Fig. S1). The same trend is also observed for 5hmC in water, with the slightly more favorable ΔG_5hmC (water)_ values of −4.8 kJ/mol for Trp, −4.2 kJ/mol for Tyr and −3.5 kJ/mol for Phe (Fig. 2a right, Table 1 and Fig. S2). On the other hand, neither CYT derivative exhibits any preference for the negatively charged side chains in water (Figs. 2a, S1 and S2, Table 1). Importantly, the amino-acid binding free energies of 5mC and 5hmC correlate with each other with a Spearman correlation coefficient ρ = 0.91 and a root-mean square deviation (RMSD) over all studied amino acids of 0.96 kJ/mol. Finally, the amino-acid free energies of both 5mC and 5hmC in water correlate closely with the corresponding binding free energies of their precursor nucleobase CYT^12^ (Table 1) with Spearman ρ_5mC-C (water)_ = 0.95 and ρ_5hmC-C (water)_ = 0.97 and RMSDs of 0.95 and 0.96 kJ/mol, respectively.

**Table 1:**
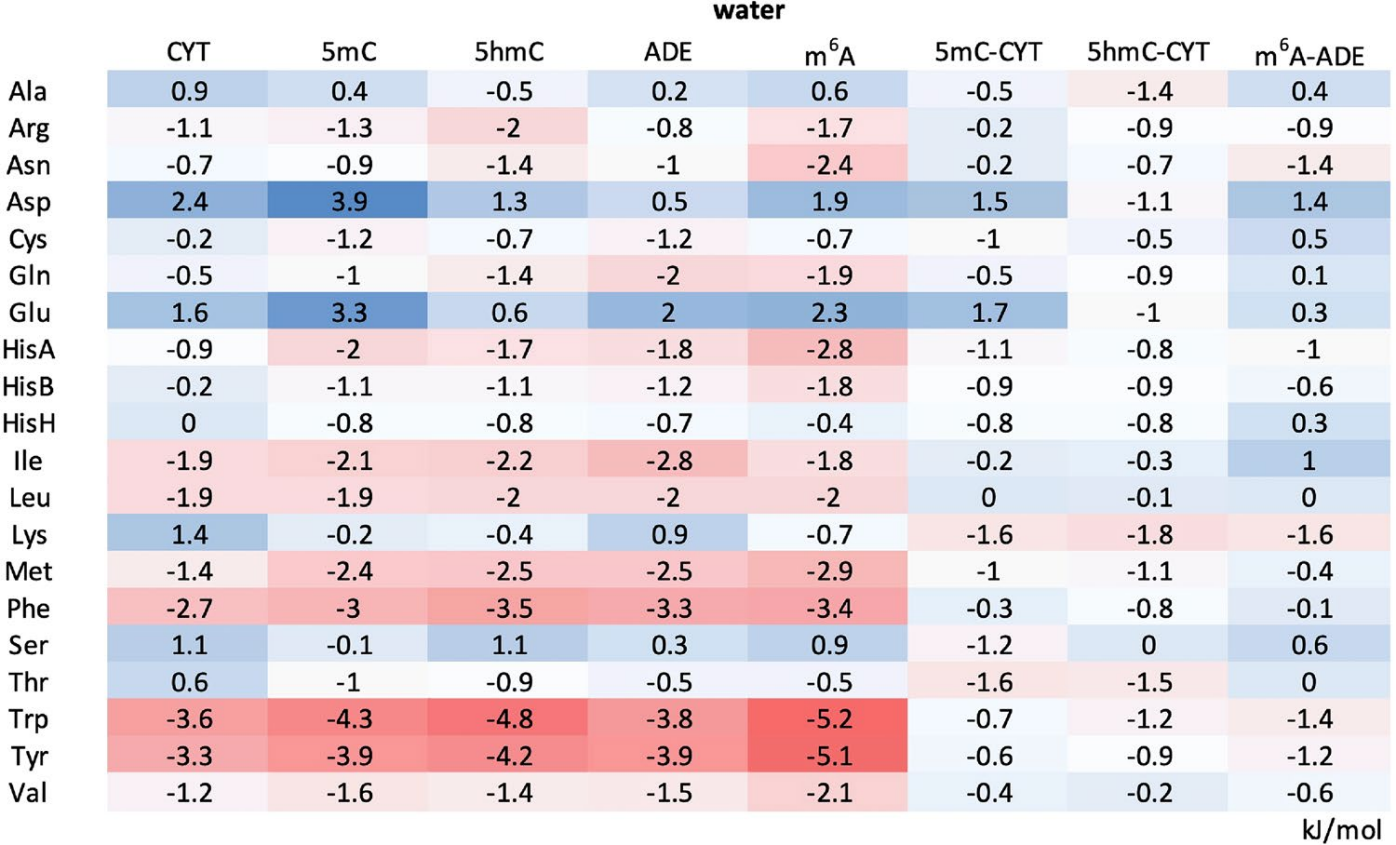
ΔG_binding_ in water between protein side chains and CYT, 5mC, 5hmC, ADE or m^6^A, together with ΔΔG_binding_ of modified nucleobases with respect to their standard counterparts.

The values for CYT and ADE were taken from de Ruiter et al.^12^. All values are given in kJ/mol.

The PMFs derived in methanol, a low-dielectric solvent meant to model the largely dehydrated RNA–protein interfaces, lead to significantly less favorable binding free energies of 5mC for most side chains as compared to those in water (Tables 1 and 2, Fig. S1). Indeed, the biggest change is observed for Ile, Tyr and Trp where the binding free energies become significantly less favorable (ΔΔG_5mC (methanol–water)_ > 3 kJ/mol). A similar behavior is observed for 5hmC, especially for Phe, Trp, Tyr and Met (Tables 1 and 2, Fig. S2). On the other hand, the binding free energies between 5hmC and the negatively charged Asp and Glu become significantly more favorable in methanol as compared to water (ΔΔG_5hmC (methanol–water)_ < −3.8 kJ/mol, Tables 1 and 2). Unlike in water, the protein sidechain affinities of 5mC and 5hmC in methanol correlate with each other only weakly (ρ_5mC-5hmC (methanol)_ = 0.18 and RMSD = 2.04 kJ/mol) with the biggest difference seen for Asp and Glu and, to a lesser degree, Phe and Trp (Fig. 2b, left). Interestingly, while the binding free energies of 5mC in methanol significantly diverge from those of CYT (ρ_5mC-CYT (methanol)_ = 0.31, RMSD_5mC-CYT (methanol)_ = 1.52 kJ/mol), the binding free energies of 5hmC in methanol are significantly more similar to those of CYT (ρ_5hmC-CYT (methanol)_ = 0.78, RMSD_5hmC-CYT (methanol)_ = 0.97 kJ/mol) (Fig. 2b, middle and right). The latter similarity stems primarily from the fact that in methanol both CYT and 5hmC interact favorably with the negatively charged side chains and unfavorably with the aromatic ones (Table 2), while with 5mC the situation is reversed. Finally, the binding free energies for the positively charged Arg and Lys side chains are the only ones where the affinities of 5hmC are more similar to 5mC than to its precursor nucleobase CYT: namely, both modified nucleobases show favorable affinities for these two side chains (Table 2).

**Table 2:**
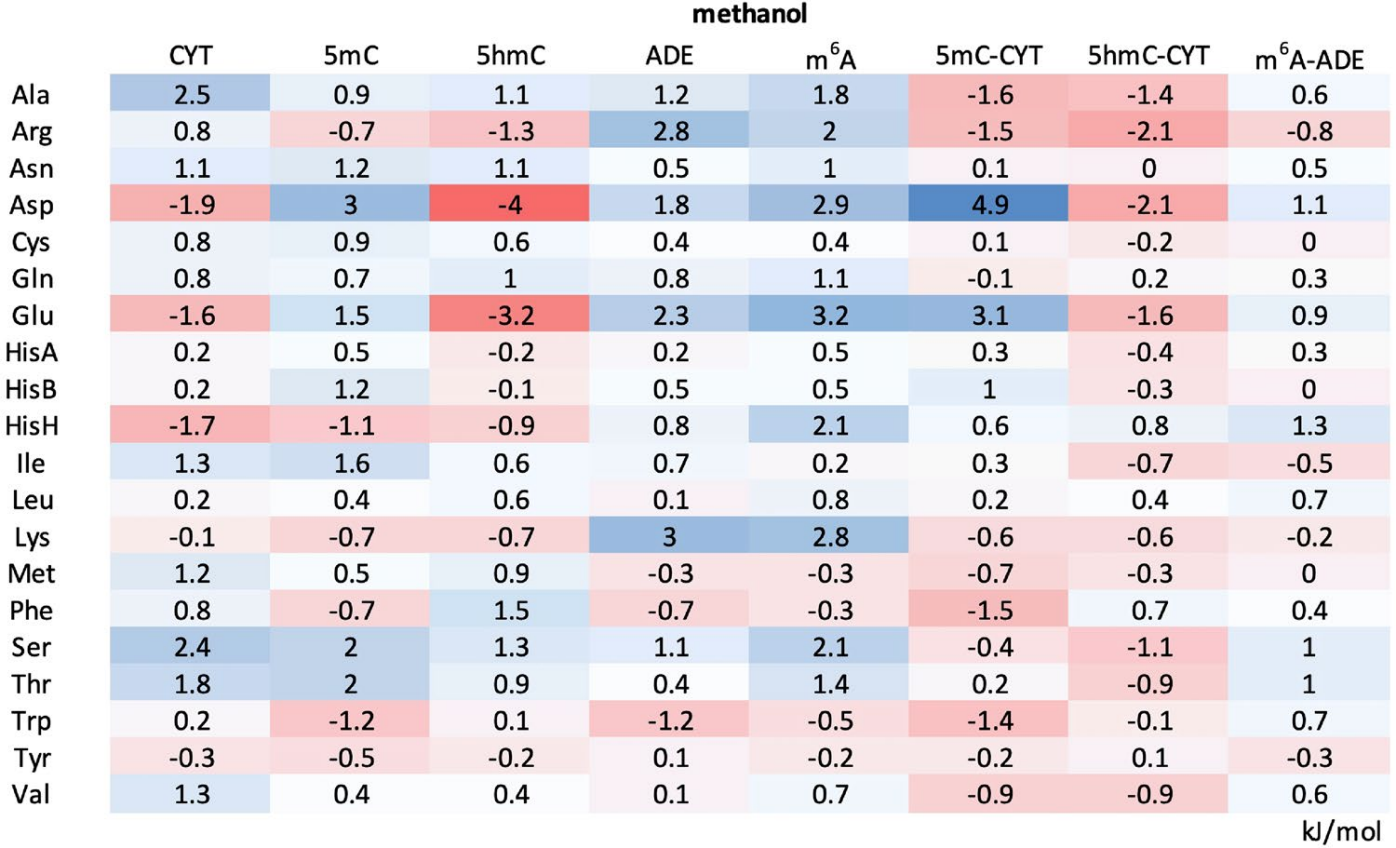
ΔG_binding_ in methanol between protein side chains and CYT, 5mC, 5hmC, ADE or m^6^A, together with ΔΔG_binding_ of modified nucleobases with respect to their standard counterparts.

The values for CYT and ADE were taken from de Ruiter et al.^12^. All values are given in kJ/mol.

### Protein sidechain binding free energies of m^6^A

**Figure 3:**
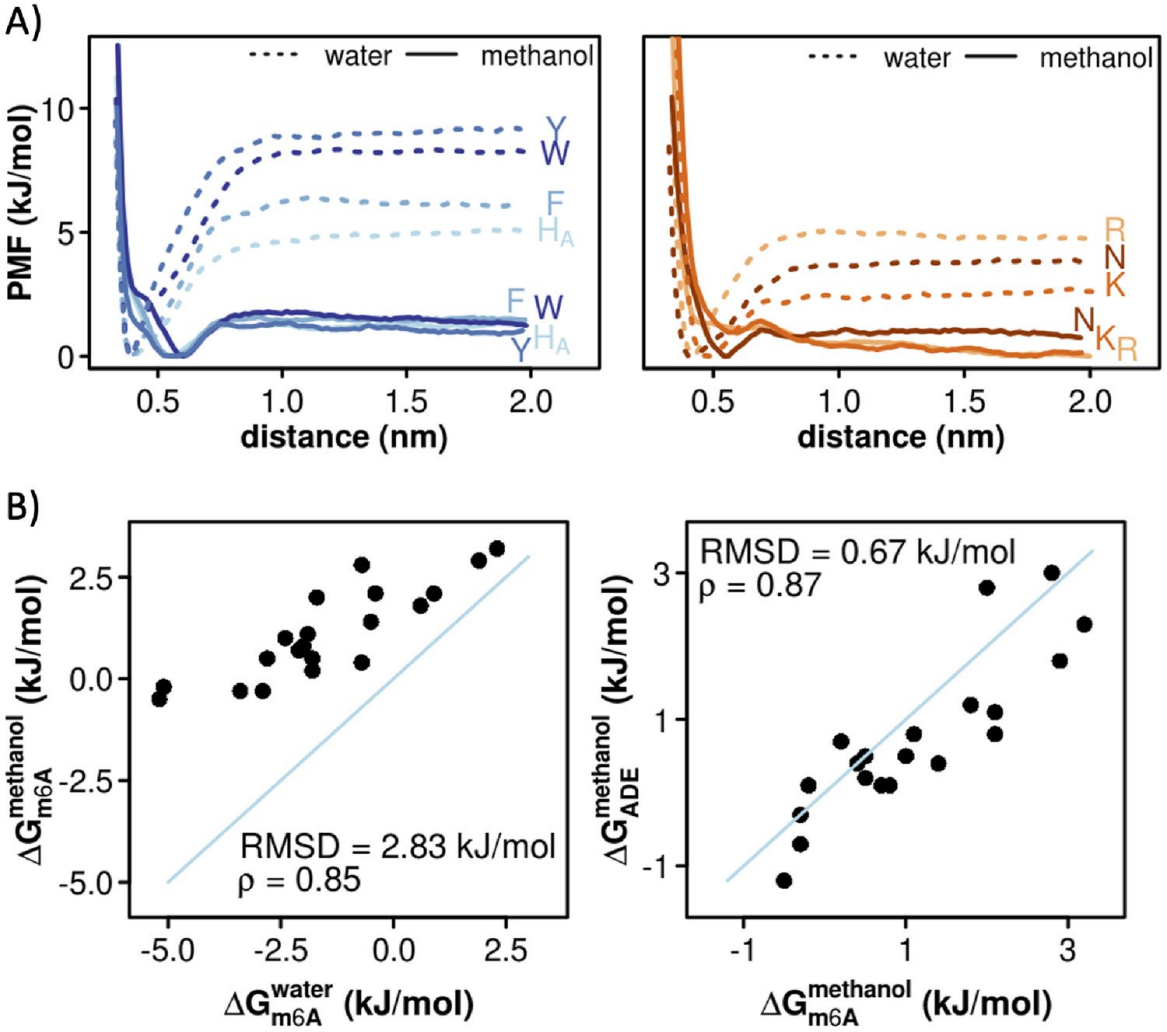
Absolute binding free energies of protein side chains for m^6^A. (**a**) PMF curves derived for protein side chains whose affinities for m^6^A change significantly (> 3.4 kJ/mol) depending on the surrounding solvent. In the left panel, affinities of aromatic side chains for m^6^A get less favorable with a decrease in the dielectric constant of the surrounding environment (from water to methanol). In the right panel, the same trend is depicted, but for polar and positively charged side chains. (**b**) A comparison between m^6^A sidechain affinities derived in water and methanol (left panel) and sidechain affinities for m^6^A and ADE^12^ derived in methanol with the corresponding Spearman correlation coefficients and RMSD values.

In water, m^6^A shows the strongest preference for aromatic side chains, with ΔG_m6A (water)_ of −5.2 kJ/mol for Trp, −5.1 kJ/mol for Tyr and −3.4 kJ/mol for Phe (Fig. 3a left panel, Table 1 and Fig. S3). Favorable interactions, albeit weaker, are also observed with other polar and non-polar protein side chains, while the unfavorable binding free energies are seen for the negatively charged ones. The same trend was also observed in a related study for ADE (Table 1) (24). In fact, the protein sidechain binding free energies of m^6^A and ADE are closely related (ρ_m6A-ADE (water)_ = 0.89 and RMSD_m6A-ADE (water)_ = 0.85 kJ/mol), with m^6^A showing slightly more favorable interactions for aromatic and positively charged residues (Table 1). On the other hand, m^6^A exhibits almost no favorable interactions with any protein side chains in methanol: for example, the binding free energy of -0.5 kJ/mol for Trp is the most favorable one of the set (Table 1, Figs. 3a and S3b). The m^6^A binding free energies derived in methanol correlate closely with those in water (ρ_m6A (methanol–water)_ = 0.85), but with a significant decrease in the binding free energies for the latter across the board (RMSD_m6A (methanol–water)_ = 2.83 kJ/mol) (Table 1 and Fig. 3b left panel). Finally, in contrast to the situation with 5mC and CYT, the m^6^A affinities in methanol are closely related to those of ADE^12^ (ρ_m6A-ADE (methanol)_ = 0.87 and RMSD_m6A-ADE (methanol)_ = 0.67 kJ/mol) (Fig. 3b right panel, Table 2).

### Convergence analysis

**Figure 4:**
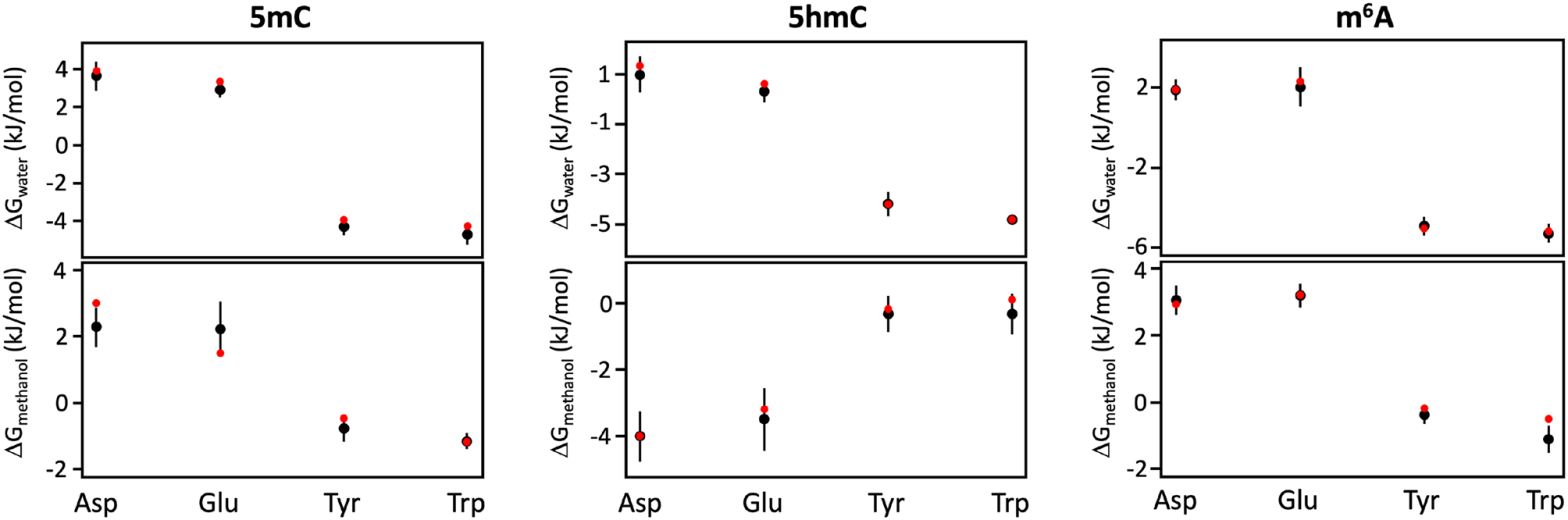
Convergence analysis. Means and standard deviations of ΔG_binding_ of select protein side chains (Asp, Glu, Tyr, Trp) for (**a**) 5mC, (**b**) 5hmC and (**c**) m^6^A, derived from 5 independent simulations runs in water and methanol. Red dots represent the values of ΔG_binding_ reported in Tables 1 and 2.

As seen above, the difference in the binding free energies of protein sidechains for modified and standard nucleobases is in some cases lower than 1.5 kJ/mol, the value chosen as the limit for the convergence of our simulations (see Methods for details). To analyze the reproducibility of our results, we have derived the absolute binding free energies of Asp, Glu, Tyr and Trp for 5mC, 5hmC and m6A in water and methanol using 5 independent simulations for each pair, starting with different initial velocities. The largest standard deviations (SDs) in ΔGs over the five replicas were seen in the case of Asp and Glu affinities for 5mC (SD_Asp_ = 0.55 kJ/mol; SD_Glu_ = 0.80 kJ/mol) and 5hmC (SD_Asp_ = 0.73 kJ/mol; SD_Glu_ = 0.92 kJ/mol) derived in methanol (Fig. 4). Regarding m^6^A affinities, the only significant discrepancy was observed for Glu in water (SD_Glu_ = 0.96 kJ/mol). In all other cases, the SDs are equal or below the 0.58 kJ/mol. Overall, this analysis suggests that our simulations exhibit reasonable convergence to within an uncertainty window of 1 kJ/mol.

### Dominance of π-based interactions in water

Stacking interactions between modified nucleobases and planar π-rings in amino acids represent some of the most dominant interaction modes observed in water, mirroring what was seen with unmodified bases before^12^. For example, such interactions are present for 50% of time or more at the PMF minima of all three modified nucleobases and all the relevant amino acids (Arg, His_A_, His_B_, Phe, Trp and Tyr) except His_H_, where this percentage drops to 20% (Table S1). The fact that the PMF minima in methanol are typically located at a larger distance as compared to water is reflected in the fact that the stacking interactions are destabilized in the former, with approximately an order of magnitude lower relative frequencies seen across the board (Table S1). This is fully analogous to the situation with unmodified precursor bases ADE and CYT, as shown before^12^. Interestingly, in the case of 5hmC interacting with Phe, Trp or Tyr, the π–π stacking remains the dominant mode of interaction also in methanol, with the relative frequencies exceeding 50% in all three cases (Table S1). We have also analyzed the cation–π and anion–π interactions in our simulations. While in the case of m6A such interactions do not contribute significantly in the case of m6A (Table S2), they are detected in a significant fraction of simulation snapshots at the PMF minima in the case of 5mC and especially 5hmC (Table S2). For example, anion–π interactions are present in 46% of all snapshots at the PMF minimum in the case of 5hmC/Asp simulations in methanol. In agreement with the lower dielectric constant of methanol as compared to water, both cation/π and anion/π interactions tend to occur more frequently in methanol simulations (Table S2).

### Hydrogen bonds with nucleobase ring edges represent a significant interaction mode in modified bases

Protein recognition of nucleobases in dsDNA or folded RNA occurs often with nucleobases being significantly more spatially restricted than in our umbrella simulations. Most importantly, the nucleobase ring planes may be inaccessible in such situations, shifting the emphasis on the recognition of ring edge patterns. In agreement with this, our simulations show strong evidence of hydrogen bonding with groups on the nucleobase ring that are accessible even in the context of dsDNA or folded RNA. Most prevalent H-bonds are seen between charged (Asp, Glu) or polar sidechains (Asn, Gln, His_A_, His_B_, Ser, Thr) and the hydroxyl group on the C5 atom and the N4 amino group of 5hmC in both water and methanol (Table S1). In addition, H-bonds are also observed between polar sidechains and the O2 group of 5hmC in methanol. A similar H-bonding pattern involving the nucleobase N4 amino group and O2 is also seen for 5mC and polar (Asn, Gln, His_A_, His_B_, Ser, Thr) and charged (His_H_, Lys, Arg) sidechains (Table S1). We do not observe any significant interactions between sidechains and the N3 group of 5mC and 5hmC, which is inaccessible in dsDNA or folded RNA (Table S1). Finally, in the case of m^6^A, the only H-bonds that are present more than 10% of simulation time are seen between N6 amino group of the nucleobase and Asn and His_A_ in methanol simulations (Table S1).

### 5mC binding free energies modulate site-specific interactions in nucleic-acid/protein complexes

**Figure 5:**
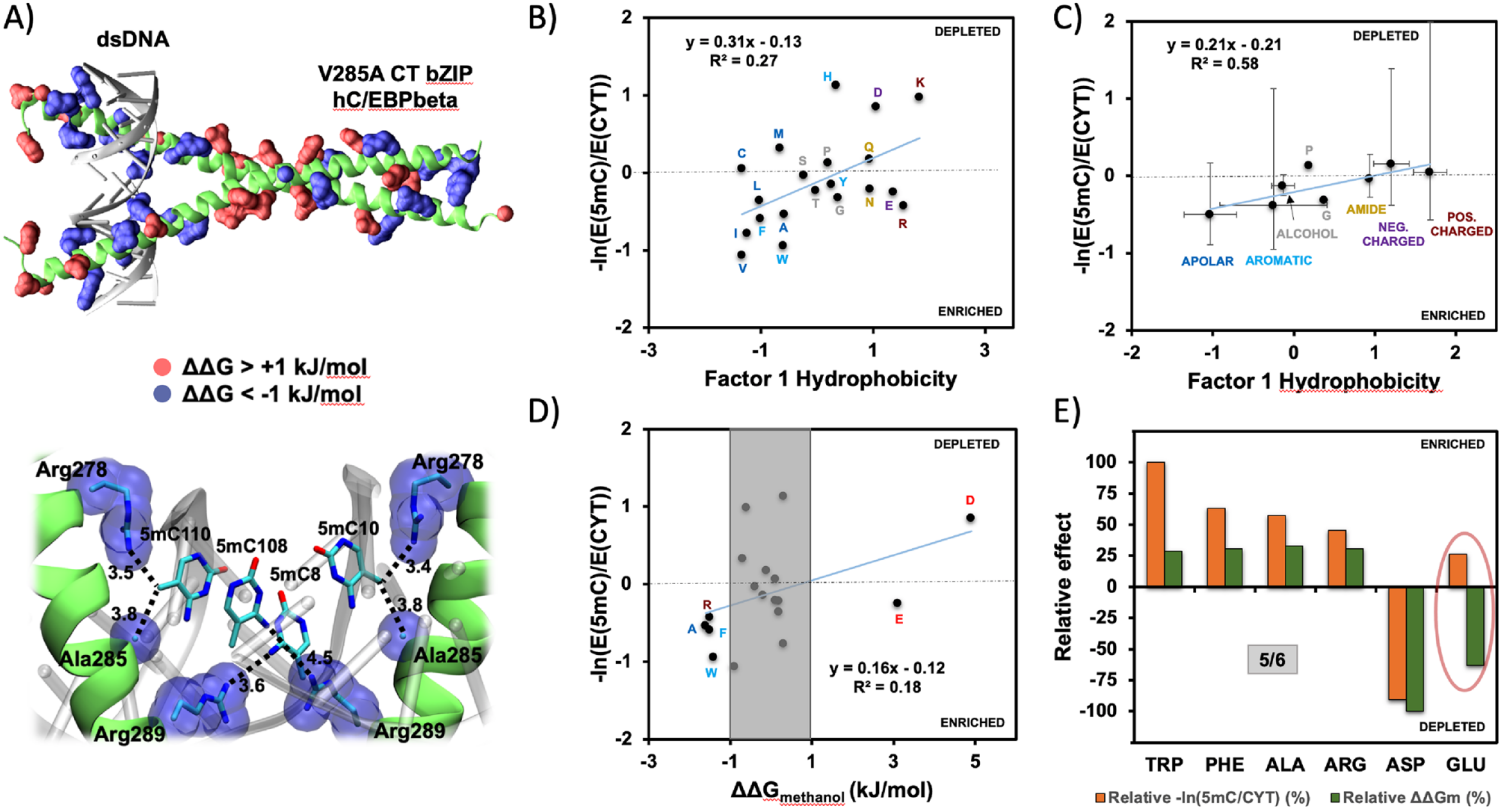
5mC sidechain binding free energies guide site-specific nucleic-acid/protein interactions. (**a**) Structure of bZIP hC/EBPβ in complex with DNA. The residues with the highest preference for 5mC over CYT in our methanol simulations are colored in blue, while the residues with the highest preference for CYT over 5mC are colored in red. (**b**) Correlation between hydrophobicities of amino acids as captured by Factor 1 scale^65^ and their relative preference for 5mC over CYT as derived from high-resolution structures of nucleic-acid/protein complexes (Pearson R^2^ = 0.27, p-value = 0.016). (**c**) Same as in (**b**), but with amino acids grouped according to their physicochemical properties (Pearson R^2^ = 0.58, p-value = 0.004). (**d**) Comparison between ΔΔG_5mC-CYT (methanol)_ for different protein sidechains and their relative preference for 5mC over CYT as derived from structural analysis (Pearson R^2^ = 0.18, p-value = 0.33). (**e**) ΔΔG_5mC-CYT (methanol)_ values and the relative preferences for 5mC over CYT as derived from structural analysis for residues with the highest relative preference for either 5mC (Trp, Phe, Ala, Arg) or CYT (Asp, Glu) in methanol simulations.

Of the three modifications studied here, the interactions of 5mC with proteins have over the years been characterized best from the structural perspective. As an illustrative example, we visualize in Fig. 5a the 3D structure of a DNA fragment in complex with the human bZIP hC/EBPβ (PDB ID: 6MG3), a transcription factor (TF) which was shown to preferentially recognize 5mC-containing DNA regions^66^. The family of basic region:leucine zipper (bZIP) DNA-binding proteins contains some of the most widely studied and best characterized TFs, which recognize related, but different palindromic DNA sequences as homodimers or heterodimers^67^. In Fig. 5a, we highlight the hC/EBPβ residues at the DNA interface, which exhibit a significant difference in their binding free energy with 5mC and CYT as derived in our methanol simulations. Our analysis shows that the residues which strongly prefer 5mC over CYT concentrate heavily at the interface between the TF and DNA, while those which prefer CYT are depleted at the interface and are enriched in the leucine zipper region of the TF (Fig. 5a). For example, Arg is heavily enriched at the interface and also exhibits one of the highest preferences for 5mC over CYT in methanol (ΔΔG_5mC-CYT (methanol)_ = −1.5 kJ/mol, Table 2). Even more significantly, the depicted variant of bZIP hC/EBPβ was optimized for 5mC binding by a V285A mutation at the DNA interface and, indeed, Ala is the residue with the highest preference for 5mC over CYT in our simulations (ΔΔG_5mC-CYT (methanol)_ = −1.6 kJ/mol, Table 2)^66^.

While suggestive, the bZIP hC/EBPβ example is clearly an isolated case. In order to probe the general applicability of the derived scales to studying the process of detecting 5mC signals in nucleic acids, we have analyzed a comprehensive set of 101 high-resolution X-ray structures of nucleic-acid/protein complexes where CYT and 5mC are seen to interact with the same protein, revealing several notable trends. First, the preference for 5mC over CYT at nucleic-acid/protein interfaces correlates with amino-acid hydrophobicity: the more hydrophobic a given residue is, the more likely it is to be found in the vicinity of 5mC as opposed to CYT (Fig. 5b,c) and vice versa. Specifically, the statistical free-energy proxy for the relative affinity of protein residues for 5mC over CYT as derived from PDB structures correlates with the Factor 1 consensus amino-acid hydrophobicity scale^65^ with a squared Pearson correlation coefficient R^2^ = 0.27 (p-value = 0.016, Fig. 5b). This correlation also significantly increases if one groups amino acids according to their chemical properties (R^2^ = 0.58, p-value = 0.004, Fig. 5c), but one should note that the data points in this case are associated with significant error bars and thus provide a mostly qualitative indication of proportionality only. What is more, the general trend when it comes to amino-acid enrichment at nucleic-acid/protein interfaces is consistent with the respective relative binding free energies (Fig. 5d). Specifically, all four residues with ΔΔG_5mC-CYT (methanol)_ < −1 kJ/mol i.e. those which exhibit a significant preference for 5mC in a low-dielectric environment (Trp, Ala, Phe, Arg), are in PDB structures also significantly enriched in the vicinity of 5mC as opposed to CYT (Fig. 5d,e). Also, the amino acid with the highest preference for CYT over 5mC in our methanol calculations, Asp, is significantly enriched in the vicinity of CYT as opposed to 5mC (Fig. 5d,e). In fact, of all the residues with a significant difference in binding ΔG with 5mC and CYT in a low-dielectric environment i.e. where |ΔΔG_5mC-CYT (methanol)_|> 1 kJ/mol (points outside of the gray area in Fig. 5d), only Glu goes against the trend predicted by ΔΔG_5mC-CYT (methanol)._ Namely, in PDB structures Glu is weakly enriched in the vicinity of 5mC as compared to CYT, although its binding free energy for CYT in methanol is more favorable by 3.1 kJ/mol (Fig. 5d,e, Table 2).

## Discussion

We have provided a detailed analysis of the absolute binding free energies between standard protein side chains and three important modified nucleobases in DNA/RNA (5mC, 5hmC and m^6^A). A comparison with the corresponding free energies of standard nucleobases derived previously^12^ suggests that select protein side are highly sensitive to nucleobase modifications. This effect is particularly pronounced for CYT and its modified derivative 5mC, where we observe a potentially relevant trend in the affinities derived in the low-dielectric environment especially for charged and, aromatic side chains (Fig. 2b). Thus, the presence of the negatively charged Asp and Glu at the interface strongly favors interactions with CYT, while the presence of positively charged Lys and Arg as well as aromatics favors interactions with 5mC. On the other hand, the protein side chain affinities of 5hmC resemble much more those of CYT than those of its precursor 5mC (Fig. 2b). This is especially relevant as 5hmC corresponds to the first intermediate in the active de-methylation process of 5mC performed by TET enzymes. When analyzed from a reductionist, physicochemical perspective, the CYT modification cycle could thus be seen as a two-step process, whereby the first modification of CYT to 5mC changes significantly the physicochemical properties of the original nucleobase, while the second step (5mC to 5hmC) almost completely restores the original physicochemical properties of CYT when it comes to interactions with protein side chains. Finally, it should be emphasized that both 5mC and 5hmC also affect the local flexibility of the nucleic acid in question as shown using single-molecule cyclization assays, together with molecular dynamics simulations^68^. The study showed that 5mC has potential to reduce the flexibility of DNA, while 5hmC appears to enhance it, which was then further linked to the nucleosome mechanical stability with the final effect on the regulation of gene expression^68^.

In contrast to CYT methylation, we do not observe a significant difference between protein sidechain affinities of ADE and its modified derivative m^6^A when it comes to the relative ordering of binding affinities (Fig. 3b). Rather, the mechanism through which m^6^A affects the interaction mode with proteins may rely more on local structural changes of DNA or RNA^46,69–71^. The methyl group of m^6^A is positioned at the border of Watson–Crick and Hoogsteen edges, which could directly influence the base-pairing with the nucleobase from the complementary strand and lead to a local structural distortion^46,69–71^. This could then be specifically differentiated from ADE by protein readers as already shown in the case of RNA^8,72^. Therefore, our results suggest that the action of the m^6^A as an epigenetic marker may primarily be based on the structural effect it exerts and likely not on the difference in the physicochemical properties when compared to its chemically modified variant. This is in contrast with ADE deamination, which results in hypoxanthine (inosine base) and significantly affects interaction preferences with protein side chains^53^. These caveats notwithstanding, it should be observed that even though the relative ordering of sidechain binding affinities does not significantly change upon ADE methylation, the ΔΔG values are still not all equal to 0. In other words, different residues still react differently to adenine methylation, a property that could in principle be used for modulating the activity of reader proteins.

In the present study, methanol was used to mimic the lower dielectric constant ε at DNA/RNA–protein interfaces. The exact value of the dielectric constant at such partially dehydrated interfaces is a local, system-specific property. For example, Alexov and coworkers have used a Gaussian dielectric model to show that the dielectric constant at DNA/RNA–protein interfaces covers a wide range going from that in protein interior to that of bulk water, depending on the exact distance between interacting atoms^73^. In particular, they showed that the average value of ε tends to be between 20 and 40 for distances between nucleic acid and protein surfaces between 1 and 2 Å (DNA) or 2.5 Å and 3.5 Å (RNA). While no model solvent can capture the full complexity of realistic nucleic-acid/protein interfaces, the usage of methanol in our study enables the analysis of the effects of the medium-range values of the dielectric constant in a controlled, reductionist manner, in addition to the advantages of methanol as being an experimentally tractable solvent. Moreover, having the binding free energies between nucleobases and amino-acid side chains at two fixed points of ε i.e. in methanol and in water, enables one to also estimate the corresponding binding free energies at all intermediate values of ε between the two limits, as demonstrated before^54^. Finally, it should be emphasized that the dielectric constant of the Gromos methanol model used in our study is 18.6, which is lower than its experimental value of 33^62^, but may actually be more relevant^61^.

We have illustrated the discriminatory power of the presently derived sidechain affinities for CYT and 5mC on the existing high-resolution structures of nucleic-acid/protein complexes (Fig. 5). We could show that the general trends in relative binding free energies determined herein largely mirror the amino-acid enrichment and depletion at the CYT an 5mC binding sites. This suggest that, at least in part, the interaction specificity between protein readers of 5mC could reside in the differences in the intrinsic affinities of protein residues for CYT and its methylated counterpart. As our results further show, these differences also depend to a degree on the dielectric properties of the local environment (Tables 1 and 2). Finally, the example of the complex between the bZIP hC/EBPβ TF and DNA illustrates a wider point that TFs not only exhibit localized hot spots that bind the modified base, but rather that their entire binding interface with DNA may contribute to the final recognition of the binding site (Fig. 5a). Indeed, the trend in the hC/EBPβ preference for 5mC suggests that the whole interface of TFs could be used to superficially scan for the methylation status of the DNA, leading ultimately to the site-specific binding of the methylated nucleobase. However, it should be strongly emphasized that the binding of nucleic acids and proteins, in addition to intrinsic nucleobase/amino-acid affinities, also depends on various factors, e.g. primary sequences^74^, 2D and 3D structures of nucleic-acid and protein binding sites^75^, cofactors^76^ and chromatin accessibility^77^. A clear evidence that other factors, such as steric hinderance, are also at play is given by the fact that hC/EBPβ does not interact preferentially with 5hmC-containing DNA^66^, although our low-dielectric scales indicate that in particular Arg residues, which feature strongly at the DNA–protein interface, should prefer 5hmC over CYT (Table 2). Finally, it should be noted that experimental crystal structures of nucleic acids are sometimes unmodified or not completely modified even in cases where modifications are expected to be detected, a fact which could skew the statistics of the relative abundancies of modified and unmodified nucleobases in our analysis.

It is our hope that this systematic study of interaction modes between three widespread modified DNA nucleobases and protein side chains with the corresponding absolute binding free energies derived in high- and low-dielectric environment will provide a better understanding of recognition of these DNA/RNA modifications by their readers, but could also assist in modulating the activity of such readers in different applied contexts.

## Supplementary Information

Supplementary Information.

## Data Availability

The datasets used and/or analyzed during the current study are available from the corresponding author on reasonable request.
