# Supplementary material for: Interaction preferences between protein side chains and key epigenetic modifications 5-methylcytosine, 5-hydroxymethycytosine and N6-methyladenine
Source: Sci Rep. 2022 Nov 15;12:19583. doi: 10.1038/s41598-022-23585-z (PMC9666514; doi:10.1038/s41598-022-23585-z)
Supplement: Supplementary file 1 — Supplementary Information. [file 41598_2022_23585_MOESM1_ESM.pdf]

Supplementary Information

Interaction preferences between protein side chains and key epigenetic modifications 5-methylcytosine, 5-hydroxymethycytosine and N^6-methyladenine

Matea Hajnic[1*], Santiago Alonso Gil[1], Anton A. Polyansky[1], Anita de Ruiter[1**] & Bojan Zagrovic[1#]

[1]Department of Structural and Computational Biology, Max Perutz Labs, University of Vienna, Campus Vienna Biocenter 5, Vienna, A-1030, Austria

[*]present address: Matea Hajnic, Bayer AG, Computational Life Sciences, Research & Development, Crop Science, Frankfurt, Germany

[**]present address: Anita de Ruiter, Institute of Molecular Modeling and Simulation, BOKU, Muthgasse 18, Vienna, A-1190, Austria

[#]to whom correspondence should be addressed. Tel: +43 1 4277 52271; Fax: +43 1 4277 9522; Email: bojan.zagrovic@univie.ac.at

PDB Codes of X-ray structures used for the analysis of contact statistics

10MH, 1BSU, 1DCT, 2C7O, 2C7P, 2C7Q, 2C7R, 2UYC, 2UYH, 2UZ4, 2ZKD, 2ZKE, 2ZKF, 2ZO0, 2ZO1, 3C2I, 3CLZ, 3F8I, 3F8J, 3FDE, 3Q0F, 3VXV, 3VXX, 3VYB, 3VYQ, 4AQU, 4AQX, 4DKJ, 4F6N, 4GJP, 4GJR, 4GZN, 4HP1, 4LG7, 4LT5, 4M9E, 4M9V, 4MHT, 4NM6, 4PW7, 4QEN, 4QEO, 4QEP, 4R2A, 4R2E, 4R2R, 4R2S, 4X9J, 5B2J, 5BT2, 5CG9, 5CPJ, 5CPK, 5EF6, 5EGO, 5J3E, 5KE7, 5KE8, 5KL4, 5KL5, 5KL7, 5MCV, 5MCW, 5MHT, 5SZX, 5T00, 5T01, 5VMU, 5VMV, 5VMW, 5VMX, 5VMY, 5VMZ, 6A5N, 6C1A, 6C1T, 6C1U, 6C1Y, 6C2F, 6CCG, 6CNP, 6D1T, 6E93, 6E94, 6JNM, 6JNN, 6JTQ, 6JVZ, 6JW1, 6JW3, 6LEW, 6M2V, 6MHT, 6ML6, 6ML7, 6R64, 6X6E, 6X9I, 6X9J, 6X9K, 6YKF, 6YMG, 7CY6, 7CY8, 7MWM.

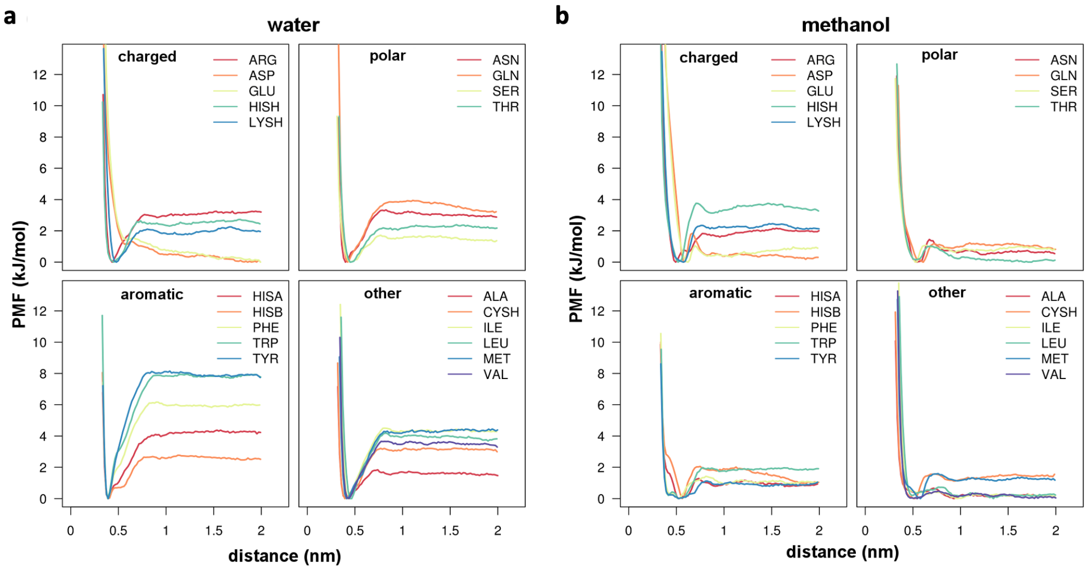

Figure S1. Potential of mean force (PMF) curves for the binding between 5mC and different amino-acid sidechain analogs derived in (**a**) water and (**b**) methanol.

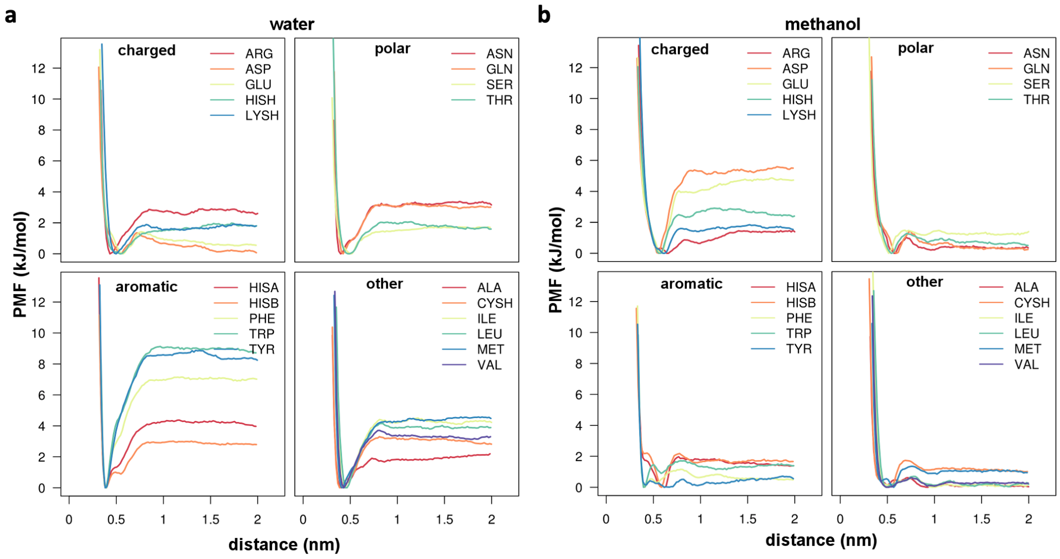

Figure S2. PMF curves for the binding between 5hmC and different amino-acid sidechain analogs derived in (**a**) water and (**b**) methanol.

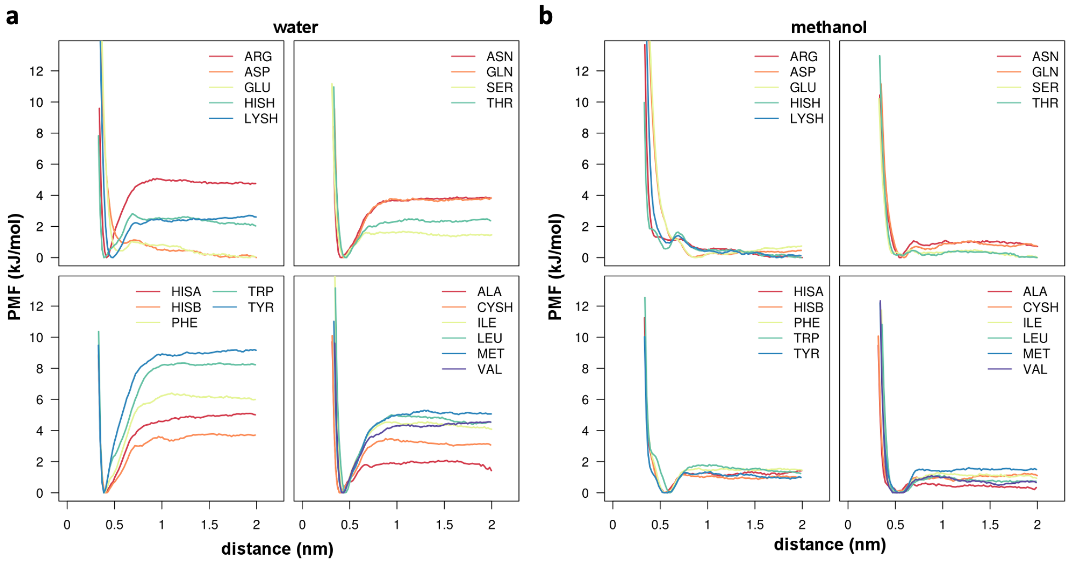

Figure S3. PMF curves for the binding between m^{6}A and different amino-acid sidechain analogs derived in (**a**) water and (**b**) methanol.

	m6A				5mC				5hmC			
	wat		met		wat		met		wat		met	
	R_0 (nm)	%	R_0 (nm)	%	R_0 (nm)	%	R_0 (nm)	%	R_0 (nm)	%	R_0 (nm)	%
**Arg**	0.4	65	0.6	5	0.4	53	0.5	11	0.4	53	0.6	2
**His_A**	0.4	58	0.5	12	0.4	61	0.5	10	0.4	63	0.6	1
**His_B**	0.4	52	0.6	2	0.4	55	0.6	1	0.4	56	0.6	1
**His_H**	0.4	60	0.6	2	0.5	20	0.5	11	0.5	22	0.6	3
**Phe**	0.4	65	0.6	2	0.4	70	0.5	19	0.4	73	0.4	58
**Trp**	0.4	76	0.6	2	0.4	82	0.5	21	0.4	85	0.4	71
**Tyr**	0.4	79	0.6	2	0.4	80	0.5	20	0.4	81	0.4	55

Table S1. Percentage of time that stacking interactions, defined[1] as the distance between the centres of geometry of the groups involved being < 0.5 nm and the angle between the planes defined by the groups involved being < 30°, are present in water (wat) or methanol (met) simulations at the window around the minimum of the PMF (R_0).

	m6A				5mC				5hmC			
	wat		met		wat		met		wat		met	
	R_0 (nm)	%	R_0 (nm)	%	R_0 (nm)	%	R_0 (nm)	%	R_0 (nm)	%	R_0 (nm)	%
cation-π												
**Lys**	0.5	2	0.6	3	0.5	13	0.6	40	0.5	10	0.6	35
anion-π												
**Asp**	0.6	10	0.6	12	0.6	6	0.6	12	0.5	29	0.5	46
**Glu**	0.5	12	0.6	9	0.6	5	0.6	10	0.6	12	0.6	28

Table S2. Percentage of time that cation-π and anion-π interactions, defined[2,3] as the distance between the centres of geometry of the ring and the charge group involved being < 0.5 nm and the angle between the ring plane and the charge group being > 50°, are present in water (wat) or methanol (met) simulations at the window around the minimum of the PMF (R_0).

	ARG		ASP		GLU		ASN		GLN		HIS$_A$		HIS$_B$	
	wat	met	wat	met	wat	met	wat	met	wat	met	wat	met	wat	met
**5mC-O2**	5	45	0	0	0	0	3	16	2	10	2	13	0	11
**5mC-N3**	3	28	0	0	0	0	3	3	1	1	0	6	0	5
**5mC-N4**	0	0	8	38	7	33	2	13	1	19	0	10	0	9
**5hmC-O2**	3	29	0	0	0	0	1	11	2	10	0	8	0	7
**5hmC-N3**	1	14	0	0	0	0	1	6	1	4	0	3	0	4
**5hmC-N4**	0	0	13	41	13	37	1	15	2	18	0	20	1	12
**5hmC-O5**	3	14	49	57	32	49	5	12	6	6	7	6	6	12
**m^{6}A-N3**	1	4	0	0	0	0	3	6	2	6	0	2	0	0
**m^{6}A-N6**	0	0	4	3	9	3	7	18	6	4	2	12	3	5
**m^{6}A-N7**	0	1	0	0	0	0	2	4	1	2	0	0	0	0

	HIS$_H$		LYS		SER		THR		TYR		TRP	
	wat	met	wat	met	wat	met	wat	met	wat	met	wat	met
**5mC-O2**	4	15	7	25	4	10	5	14	0	8	0	5
**5mC-N3**	3	9	4	11	1	2	1	3	0	2	0	3
**5mC-N4**	0	0	0	0	4	12	2	10	0	0	0	0
**5hmC-O2**	2	11	6	19	3	11	3	9	0	2	0	0
**5hmC-N3**	2	6	3	7	1	2	1	2	0	0	0	0
**5hmC-N4**	0	0	0	0	7	14	4	18	0	1	0	0
**5hmC-O5**	2	4	3	6	5	13	4	11	0	4	0	1
**m^{6}A-N3**	0	0	0	0	3	0	0	0	0	0	0	0
**m^{6}A-N6**	0	0	0	0	5	1	2	1	0	4	0	0
**m^{6}A-N7**	0	0	0	0	2	0	0	0	0	0	0	0

Table S3. Percentage of time that a given H-bond, defined[4] by a minimum donor-hydrogen-acceptor angleof 135° and a maximum hydrogen-acceptor distance of 0.25 nm, is present in water (wat) or methanol (met) simulations at the window around the minimum of the PMF.

REFERENCES

1. de Ruiter, A. and Zagrovic, B. (2014) Absolute binding-free energies between standard RNA/DNA nucleobases and amino-acid sidechain analogs in different environments. *Nucleic Acids Res.*, **43**, 708–718.

2. Lucas, X., Bauza, A., Frontera, A. and Quinonero, D. (2016) A thorough anion–pi interaction study in biomolecules: on the importance of cooperativity effects. *Chem. Sci.*, 7, 1038.

3. Gallivan, J. P. and Dougherty, D. A. (1999) Cation-π interactions in structural biology. *PNAS*, 96, 9459-9464.

4. van Gunsteren, W. F., Billeter S. R., Eising A. A., Hünenberger, P. H., Krüger, P., Mark, A. E., Scott, W. R. P. and Tironi, I. G. (1996) Biomolecular simulation: the GROMOS96 manual and user guide. VdF: Hochschulverlag AG an der ETH Zürich.
